# Retraction Note: LncRNA PTCSC3 is upregulated in osteoporosis and negatively regulates osteoblast apoptosis

**DOI:** 10.1186/s12920-024-01875-x

**Published:** 2024-04-17

**Authors:** Xingchao Liu, Mingliang Chen, Qinghe Liu, Gang Li, Pei Yang, Guodong Zhang

**Affiliations:** 1 Orthopedics Department, Hebei Yanda Hospital, Yanjiao Development Zone, 065201 Sanhe City, Langfang City, Hebei Province People’s Republic of China; 2 Orthopedics Department, Beijing Chao-Yang Hospital, Capital Medical University, No 8 Gongtinan Road, 100020 Beijing, People’s Republic of China

**Retraction Note: BMC Medical Genomics (2022) 15:57**

10.1186/s12920-022-01182-3

The Editors have retracted this article because of a significant overlap with an unpublished manuscript by different authors that was previously submitted to a different journal. The authors did not respond to requests for clarification nor to correspondence about this retraction.

